# Could do better! A high school market survey of fish labelling in Sydney, Australia, using DNA barcodes

**DOI:** 10.7717/peerj.7138

**Published:** 2019-06-14

**Authors:** Andrew Mitchell, Anna Rothbart, Greta Frankham, Rebecca N. Johnson, Linda E. Neaves

**Affiliations:** 1 Australian Museum Research Institute, Australian Museum, Sydney, NSW, Australia; 2 Sydney Grammar School, Darlinghurst, NSW, Australia; 3 Royal Botanic Garden Edinburgh, Edinburgh, Scotland, UK

**Keywords:** DNA barcoding, Citizen science, Fish, Fisheries, Education, High school, Seafood, Labelling, Fraud, Sustainability

## Abstract

**Background:**

Processed seafood products are not readily identifiable based on physical characteristics, which leaves the industry vulnerable to high levels of product mislabelling (globally estimated at 5–30% mislabelled). This is both a food safety issue and a consumer protection issue as cheaper species could be substituted for more expensive species. DNA barcoding is proving to be a valuable tool for authentication of fish products. We worked with high school students to perform a market survey and subsequent species assessment via DNA barcoding to investigate the accuracy of fish product names used by retailers in Sydney, Australia.

**Methods:**

Sixty-eight fish samples, sold under 50 different common names, were purchased anonymously from two retailers in Sydney. Each product name was recorded and reconciled with the Australian Fish Names Standard (AFNS). Samples were DNA barcoded and resulting sequences were deposited in the online Barcode of Life Data system using the simplified Student Data Portal interface.

**Results:**

Forty percent of the fish names did not comply with the AFNS, however, half of these were either spelling errors or vendors supplied more information than the standard requires. The other half of the non-compliant samples were given common names not listed on the AFNS. Despite this lack of standardization, DNA barcode data confirmed the retailers’ identifications for 93% of samples and 90% of species sampled.

**Discussion:**

The level of mislabelling we report for Sydney retailers (7% of samples or 10% of species) compares favorably with the global rates of 5–30%, but unfavorably with the only previous DNA barcode fish authentication study for Australia, which found no confirmed mislabelling in Hobart. Our study sampled mostly Australian produce, only two retailers and no restaurants. Results of our limited sample suggest that although many Sydney fish retailers attempt to implement the voluntary fish name standards, the standards are inadequate. As Australia imports 75% of its seafood, and in other countries restaurants generally show lower levels of compliance than retailers, broader surveys are needed before generalizing these results. DNA barcoding is a powerful yet simple method supported by accessible online analytical tools. Incorporation of fish barcoding into high school science classes provided students with valuable firsthand experience in scientific research and drew together different strands of the NSW curriculum relating to genetics and sustainability. Given the techniques, equipment, and reagents are now readily accessible, we expect to see greater uptake of DNA barcoding technology by high schools, citizen scientists and consumer groups in Australia in future. However, there remains much scope for further development of DNA barcode diagnostics (both data and analytical methods) for commercial fish species.

## Introduction

Accurate identification of fish species is necessary for sustainable management of fisheries and for consumer protection. Fish species perceived as more palatable typically sell at many times the price of less desirable species, yet once fish are processed into fillets and other products they lack the morphological attributes needed for species identification. This coupling of financial incentives (‘motivation’) with a low risk of detection (‘opportunity’) creates an ideal environment for fraudulent labelling of fish sold to the public, according to the iconic Fraud Triangle theory (Schuchter & Levi, 2016).

The issue of fish labelling laws has seen wide media coverage in Australia in recent years. The Australian Fish Names Standard (AFNS) AS SSA 5300-2015 (hereafter AFNS) recognises that standard names are needed for accurate trade descriptors, effective fisheries monitoring and management, sustainability of fisheries resources, effective traceability, food safety management and public, and consumer confidence. However, the AFNS is a voluntary standard and there is little empirical evidence of the degree to which this voluntary standard is upheld, because the historical difficulty in identifying processed fish products has prevented a rigorous assessment of compliance.

DNA-based methods are being used increasingly around the world for seafood species identification (Pollack et al., 2018; Guardone et al., 2017). One method in particular, DNA barcoding, has gained wide acceptance for identifying processed fish products. DNA barcoding is the compilation of a reference database of short DNA sequences that are usually diagnostic for species, and its subsequent interrogation to identify unknown samples (Hebert et al., 2003). The Barcode of Life Data System (BOLD; Ratnasingham & Hebert, 2007) contains some 192,000 publicly available sequences from more than 16,000 fish species (accessed 27 November 2018). The United States Food and Drug Administration has accepted the use of DNA barcoding for fish authentication (Yancy et al., 2008) and has published reference sequences for commercial species and protocols for using DNA barcoding (Handy et al., 2011). In May 2016 the European Parliament passed resolution 2016/2532 (RSP) on traceability of fish products ‘call(ing) on the Commission to exploit the potential of DNA barcoding, which could assist in the identification of species by DNA sequencing, in order to enhance traceability’.

Despite being a relatively young discipline compared to traditional morphological taxonomy, DNA barcoding technology has progressed rapidly and the barriers to entry are low: DNA extraction and polymerase chain reaction (PCR) are easily accomplished by any modestly equipped laboratory, DNA sequencing of PCR products can be outsourced to commercial services, and all the analytical tools necessary for assembly, editing and analysis of the resulting DNA sequence data are freely available online. Indeed, the first seafood market survey using DNA barcoding was performed by two high school students in New York City and received widespread press coverage when high levels of fish substitution were detected in sushi restaurants in New York (Wong & Hanner, 2008).

More than 50 DNA barcode-based seafood market surveys were performed across six continents in the 5 years up to 2016 (Pardo, Jiménez & Pérez-Villarreal, 2016) and such studies are increasingly common. Restaurants generally have been found to have higher rates of mislabelling than fishmongers and supermarkets (Vandamme et al., 2016). There is much variation in mislabelling rates, with Europe originally reporting error rates of around 30% but more recently as low as 5% (Mariani et al., 2015). A recent study in Canada reported mislabelling rates of 25% in Vancouver (Hu et al., 2018), while for studies focused on species previously found to be mislabelled, it is as high as 76% (De Brito et al., 2015). Apart from the obvious economic effects for consumers, there may be food safety concerns. For example, Armani et al. (2015) identified two samples of ‘squid’ as a poisonous pufferfish banned from sale in the EU, and accurate identification of tuna species is necessary if one wants to limit mercury intake (Mitchell & Hellberg, 2016).

Only one DNA barcode-based survey conducted in Australia has been published in the scientific literature to date: Lamendin, Miller & Ward (2015) sampled 51 fresh and unprocessed fish products from 15 retailers in Hobart, Tasmania, and obtained DNA barcode data for 38 samples. Unusually, they found no cases of mislabelling with all samples either accurately matching the expected sequences on BOLD, matching multiple congeneric species, or being unidentifiable because the database lacked the appropriate reference sequences.

In this study we performed the second Australian DNA barcode-based fish authentication survey as a citizen science project, a collaboration between a High School (Sydney Grammar School) and a scientific research institute (Australian Museum). Our major aim was to assess the potential for integrating teaching of different aspects of the biology national curriculum (e.g. sustainability and genetics) while also generating preliminary scientific data on the accuracy of species labelling for seafood in the marketplace in Sydney, Australia. We also aimed to address three separate issues relating to standard names that warrant further investigation in Australia: (1) Do sellers label fish species correctly according to the standard (AFNS), that is, are the correct standard (common) names being applied to any given species? (2) Are fish species being correctly identified? (3) Are the AFNS names adequate to achieve the stated aims of the standard while gaining an accurate understanding of fish for sale in Australia?

## Materials and Methods

### Sampling

We aimed to sample as many of the different species of fish available locally as possible. In total we purchased 68 different fish or fish products, sold under 50 different common names (Table 1). Initially, three frozen fish products were purchased from a supermarket and used to optimize the analytical protocols. Subsequently we purchased two batches of approximately 30 samples each from two retailers at Sydney Fish Market and from a fourth retailer in the Sydney central business district. The sampling was conducted in 2015–2016. Each batch consisted mostly of fresh fish fillets with only four whole fish in each batch.

**Table 1:** Samples.

BOLD sample ID	BOLD process ID	GenBank accession	Retailer name (sold as)	Australian fish names standard (AS 5300-2015) (if different)	Australian fish names standard (AS 5300-2015) species	BOLD identification (if different from AFNS species)	BOLD match %
SGS005	SDP331073-16	KX781935	Atlantic Salmon		*Salmo salar*		100
SGS007_2015	SDP331074-16	KX781943	Atlantic Salmon		*Salmo salar*		100
SGS010	SDP331015-15	KX781869	Atlantic Salmon		*Salmo salar*		100
SGS226	SDP331055-16	KX781876	Atlantic Salmon		*Salmo salar*		100
SGS003_2015	SDP331072-16	(none)	Barramundi		*Lates calcarifer*		100
SGS107	SDP331027-16	KX781875	Barramundi		*Lates calcarifer*		100
SGS217	SDP331065-16	KX781858	Barramundi		*Lates calcarifer*		100
SGS011-2015	SDP331018-15	KX781855	Basa		Pangasiidae	*Pangasianodon hypophthalmus*	100
SGS206	SDP331069-16	KX781890	Big eye ocean Perch	Bigeye ocean Perch	*Helicolenus barathri*	*H. barathi, H. lahillei, H. dactylopterus*	99.08
SGS125_2016	SDP331035-16	KX781857	Bigeye ocean Perch		*Helicolenus barathri*	*H. barathi, H. lahillei, H. dactylopterus*	100
SGS118_2015	SDP331081-16	KX781945	Blue eye Trevalla	Blue-eye Trevalla(s)	*Hyperoglyphe antarctica* & *Schedophilus labyrinthica*	*H. antarctica*	100
SGS221			Blue eye Trevalla	Blue-eye Trevalla(s)	*Hyperoglyphe antarctica* & *Schedophilus labyrinthica*	(No data)	n/a
SGS214	SDP331085-16	KX781937	Blue Grenadier		*Macruronus novaezelandiae*	*M. novaezelandiae, M. magellanicus*	100
SGS204	SDP331054-16	KX781882	Blue Mackerel		*Scomber australasicus*		100
*SGS108-2016	SDP331039-16	KX781873	Blue Warehou		*Seriolella brama*	*S. punctata, S. porosa*	100
SGS102_2016	SDP331075-16	KX781940	Bluespotted Goatfish		*Upeneichthys vlamingii*	*U. vlamingii, U. lineatus*	
*SGS209	SDP331047-16	KX781853	Bream	(NOT on list)	Eight spp. of Sparidae on commercial list	*Nemadactylus macropterus, N. bergi*	99.36
SGS203	SDP331042-16	KX781885	Crimson Snapper		*Lutjanus erythropterus*		99.85
SGS119	SDP331030-16	KX781864	Deep sea Perch	Orange Roughy	*Hoplostethus atlanticus*		100
SGS104			Flathead		Platycephalidae—undifferentiated	(No data)	n/a
SGS223			Flathead		Platycephalidae—undifferentiated	(No data)	n/a
*SGS117	SDP331080-16	KX781946	Flathead		Platycephalidae—undifferentiated	*Chelidonichthys kumu, C. spinosus*	99.21
SGS101			Garfish		Hemiramphidae—undifferentiated	(No data)	n/a
SGS225	SDP331071-16	KX781861	Goldband Snapper		*Pristipomoides multidens, P. typus*	*P. multidens, P. maculatus*	100
SGS230	SDP331063-16	KX781872	Grenadier	Blue Grenadier	*Macruronus novaezelandiae*	*M. novazelandiae, M. magellanicus*	100
SGS109	SDP331077-16	KX781933	Hoki		*Macruronus* spp.	*M. novazelandiae, M. magellanicus*	99.69
SGS126	SDP331025-16	KX781868	Hoki		*Macruronus* spp.	*M. novazelandiae, M. magellanicus*	100
SGS123_2016	SDP331037-16	KX781921	John Dory		*Zeus faber*		100
SGS216_2016	SDP331087-16	KX781934	John Dory		*Zeus faber*		100
SGS234	SDP331060-16	KX781892	King Trout	Trout	*Oncorhynchus mykiss*		100
SGS111	SDP331032-16	KX781877	Kingfish	Yellowtail Kingfish	*Seriola lalandi*	*S. lalandi, S. zonata*	100
*SGS115	SDP331029-16	KX781881	Lachet (*sic*)		*Pterygotrigla polyommata*	*Chelidonichthys kumu, C. spinosus*	100
SGS113_2015	SDP331078-16	KX781939	Ling		*Genypterus* spp.	*G. blacodes*	100
SGS208	SDP331064-16	KX781874	Mahi-mahi	Mahi Mahi(s)	*Coryphaena* spp.	*C. hippurus*	100
SGS212	SDP331066-16	KX781884	Marlin		Istiophoridae—undifferentiated	*Kajikia audax, K. albida*	100
SGS122			Monkfish	(NOT on list)		(No data)	n/a
SGS215	SDP331086-16	KX781931	Monkfish/Stargazer	Stargazer	Uranoscopidae—undifferentiated	*Kathetostoma giganteum*	100
SGS129	SDP331040-16	KX781883	Monkfish/Stargazier (*sic*)	Stargazer	Uranoscopidae—undifferentiated	*Kathetostoma giganteum*	100
SGS232	SDP331067-16	KX781850	Mt Cook Alpine Salmon	Salmon	*Oncorhynchus* & *Salmo* spp.	*Oncorhynchus tshawytscha*	100
SGS116	SDP331034-16	KX781880	Ocean Jacket		*Nelusetta ayraudi*		100
SGS128	SDP331084-16	KX781942	Ocean Perch		*Helicolenus barathri* & *Helicolenus percoides*	*H. barathi, H. lahillei, H. dactylopterus*	100
SGS124	SDP331023-16	KX781859	Ocean Trout	Trout	*Oncorhynchus mykiss* & *Salmo trutta*	*Oncorhynchus mykiss*	100
SGS210	SDP331053-16	KX781852	Ocean Trout	Trout	*Oncorhynchus mykiss* & *Salmo trutta*	*Oncorhynchus mykiss*	100
SGS121	SDP331028-16	KX781924	Orange Roughy		*Hoplostethus atlanticus*		100
SGS207	SDP331059-16	KX781856	Orange Roughy		*Hoplostethus atlanticus*		100
SGS127	SDP331083-16	KX781941	Oreodory	Smooth Oreodory	*Pseudocyttus maculatus*		98.6
SGS205	SDP331051-16	KX781854	Pink Ling		*Genypterus blacodes*		100
SGS222	SDP331052-16	KX781862	Pink Ling		*Genypterus blacodes*		100
SGS218			Pink Snapper	Snapper	*Pagrus auratus*	(No data)	n/a
SGS106	SDP331036-16	KX781867	Rainbow Trout	Trout	*Oncorhynchus mykiss*		100
SGS231	SDP331058-16	KX781849	Redfish		*Centroberyx affinis*	*C. affinis, C. australis*	100
SGS114	SDP331079-16	KX781944	Ribaldo	Deepsea Cod(s)	*Mora moro* & *Lepidion* spp.	*Mora moro*	100
SGS105	SDP331026-16	KX781866	Salmon	Atlantic Salmon	*Salmo salar*		100
SGS202	SDP331056-16	KX781923	Sand Whiting		*Sillago ciliata*	*Sillago ciliata, S. analis*	100
SGS233_2016	SDP331088-16	KX781932	Sea Garfish	Eastern Sea Garfish	*Hyporhamphus australis*	*H. australis, H. melanochir, H. ihi, Arrhamphus sclerolepis*	100
SGS201	SDP331046-16	KX781851	Sea Mullet		*Mugil cephalus*		99.69
SGS229	SDP331068-16	KX781863	Shark	(NOT on list)	(13 specific sharks on commercial spp. list)	*Carcharhinus brevipinna, C. limbatus*	100
SGS228			Snapper		*Pagrus auratus*	(No data)	n/a
SGS213	SDP331050-16	KX781889	Sockeye Salmon	Salmon	*Oncorhynchus nerka*		100
SGS120	SDP331082-16	KX781936	Spanish Mackerel		*Scomberomorus commerson*		100
SGS219	SDP331043-16	KX781860	Striped Marlin		*Tetrapturus audax*	*Kajikia audax, K. albida*	100
SGS110-2016	SDP331031-16	KX781878	Swordfish		*Xiphias gladius*		100
SGS220	SDP331070-16	KX781925	Swordfish		*Xiphias gladius*		100
SGS211	SDP331044-16	KX781865	Yellow Belly Flounder	Yellowbelly Flounder	*Rhombosolea leporina*		100
SGS103	SDP331076-16	KX781938	Yellow Tail Scad	Yellowtail Scad	*Trachurus novaezelandiae*	*T. novaezelandiae, T. japonicus, T. declivis*	99.85
SGS227	SDP331061-16	KX781922	Yellow Tail Scad	Yellowtail Scad	*Trachurus novaezelandiae*	*T. novaezelandiae, T. japonicus, T. declivis*	99.85
SGS112	SDP331024-16	KX781891	Yellowfin Tuna		*Thunnus albacares*	*T. albacares, T. atlanticus, T. obesus*	100
SGS224	SDP331062-16	KX781879	Yellowfin Tuna		*Thunnus albacares*	*T. albacares, T. atlanticus, T. obesus*	100

**Note:**

Asterisk preceding Sample ID indicates a misidentified sample. The BOLD Identification column lists all the species suggested by BOLD as possible matches and is left blank if the BOLD identification matches the name(s) listed in the Australian Fish Names Standard (previous column). The BOLD match percentage is for the closest match.

### Label analysis

For each fish sample we recorded in Table 1 the name provided by the retailer. If this name was not listed on the AFNS, we then inferred the name the retailer had meant to apply, for example, if there were spelling errors, or the retailer used an obsolete name, or the retailer failed to provide enough detail (e.g. using ‘Grenadier’ instead of ‘Blue Grenadier’) and recorded the inferred AFNS name in the next column. Label analysis was based on the comparison of names in these three columns, counting matches, mismatches, and inferring the possible reasons for mismatches.

### Laboratory protocols

#### DNA extraction: optimization and final protocol

Initially, museum staff provided short training sessions in all laboratory techniques prior to commencement, including sterile technique, tissue sampling, use of pipettes, etc. Then, wet laboratory work (DNA extraction and PCR amplification) was carried out in the high school science classroom by the 14 participating students under the supervision of school and museum staff.

Approximately 20 mg of tissue was removed from each fish sample using a sterile scalpel blade and placed into 1.5 mL microcentrifuge tubes. To better fit with school scheduling, rapid DNA extraction processes were used. Initial trials on 12 samples from three fish products used a chelex-based DNA extraction protocol (Walsh, Metzger & Higuchi, 1991). As this protocol requires very accurate pipetting, it was successful for less than half the samples carried out by the students, thus we subsequently switched to the Bioline MyTaq™ Extract-PCR Kit (Bioline, Alexandria, Australia). The Bioline MyTaq™ Extract-PCR Kit appeared more robust to use by non-experts and was used for all subsequent samples following the manufacturer’s recommended protocols. However, we also compared the recommended protocol of diluting the final supernatant DNA solution 10-fold in water, vs. not diluting, finding that both methods were effective. Five successful samples from the optimization stage were included in the final data set (sample numbers SGS005–SGS011).

#### PCR amplification and sequencing

Polymerase chain reaction was performed on a Perkin Elmer 2400 PCR machine in the classroom. PCR used 40 cycles in 25 μL volumes following the manufacturer’s recommended protocols, with a final MgCl_2_ concentration of 2.5 mM and annealing temperatures of 50–52 °C. Degenerate PCR primers were designed for this study based on a published primer cocktail. We took two published forwards primers, VF2_t1 and FishF2_t1 (Ivanova et al., 2007), which bind to exactly the same region of the COI gene but have different nucleotides in some positions, took the consensus sequence of these primers to make a degenerate sequence, and added a 5′-M13 tail to obtain our primer AMFishFm (5′-*GTAAAACGACGGCCAGT*CRACYAAYCAYAAAGAYATYGGCAC-3′). We repeated this procedure for the two reverse primers VF2_t1 and FishF2_t1 to obtain the degenerate reverse primer AMFishRm (5′-*CAGGAAACAGCTATGAC*ACYTCAGGGTGWCCGAARAAYCARAA-3′). The nucleotides in italics are 5′-M13 tails, used to facilitate sequencing with primers M13F (−21) and M13R (−27) (Messing, 1983). All primers are registered in the BOLD primer database. We also compared the performance of our newly designed primers with the primer pair FishF1/FishR1 (Ward et al., 2005).

Polymerase chain reaction products were visualised in the classroom using the E-Gels Precast Agarose Gel system (ThermoFisher Scientific, Waltham, MA, USA), and amplified PCR products were either sent to Macrogen (Seoul, South Korea) for Exo-SAP purification and DNA sequencing on an ABI 3730XL or were purified using ExoSAP-IT (Thermo Fisher Scientific, Scoresby, Victoria, Australia) in the Australian Centre for Wildlife Genomics, Australian Museum, and sequenced by the Australian Genome Research Facility (Sydney, Australia).

#### Sample replication

To test for consistency among students, we replicated sampling for 10 samples on different days and performed separate DNA extractions, PCRs and DNA sequencing. For 34 additional samples (sample IDs SGS201–SGS234) we performed PCR using both primer pair AMFishFm/AMFishRm and primer pair FishF1/FishR1. Sequencing results were compared. Only the highest quality forwards and reverse direction trace files were uploaded to BOLD for each sample.

#### Sequence upload and comparison

The BOLD student data portal (SDP) (Santschi et al., 2013) was used for initial analyses performed by the students. For samples with high quality sequence trace files in both forward and reverse directions, trace files were uploaded to the SDP, automatically assembled, and manually edited when necessary. The resulting consensus sequences were then entered into the SDP. All trace files were also edited and assembled using Geneious 9.1 (Kearse et al., 2012), and the resulting consensus sequences were used in BOLD searches on July 14, 2016, using the Species Level and Public Record databases on BOLD. Consensus sequences were uploaded to BOLD SDP if there was not already a consensus sequence in BOLD for that sample.

## Results

### Sampling

Table 1 provides details for each of the 68 samples of the name it was sold under and the corresponding common names and species names in the AFNS (AS SSA 5300-2015). DNA barcode data was obtained for 61 of 68 samples (90%).

### Label analysis

Compliance with AFNS (AS SSA 5300-2015) was assessed for all 68 samples. Of the 50 common names used, only 30 names, or 60%, agreed completely with the AFNS.

- Five names differed from the Standard Name only in spelling and the intention was obviously to use the correct name, for example, Yellow Tail Scad vs. Yellowtail Scad.
- Four names differed because they provided more information than required by the standard, for example, Sockeye Salmon vs. Salmon, Pink Snapper vs. Snapper.
- Six names were shortened versions of the correct name, and did not provide sufficient information to identify the correct species, for example, Oreodory vs. Smooth Oreodory, Sea Garfish vs. Eastern Sea Garfish.
- Two names were obsolete, that is, Ribaldo was used instead of Deepsea Cod and Deep Sea Perch was used instead of Orange Roughy.
- Three names used by retailers, Monkfish, Shark, and Bream, are not listed in the Australian Standard at all. Monkfish are species of the genus *Lophius* (Lophiidae) and are not found in Australian waters. Species of the similar looking Stargazer family (Uranoscopidae) are sometimes sold as Monkfish in Australia. Shark could be any of over 100 species, while Bream could be any of at least eight species such as Black Bream or Frypan Bream.

Taking the above points into account and substituting the correct Standard Name for each fish we found that the 68 samples were represented by 43 Standard Names. Of these names, 27 refer to single species and 16 refer to groups of species, although four of the group names are not unique, referring to overlapping sets of species groups. Thus, there were only 39 unique names to test with DNA barcode data. These are reflected in Table 1.

### Sequence upload and comparison

Table 1 lists the BOLD Sample ID, Process ID, and GenBank Accession numbers for each DNA sequence. The complete DNA alignment, sequence trace files and specimen images can be viewed on BOLD as published project SDP331.

As seen in Table 1, DNA barcode data either confirmed or did not contradict the labelled identifications of all but 4 of 61 samples and 39 standard names, that is, 93% of samples and 90% of standard names were correctly labelled.

DNA barcodes appear to provide species-level discrimination for all but four species. For *Kajikia audax*, *Macruronus novaezelandiae*, and *Sillago ciliata*, BOLD indicated 100% matches to two species and for *Helicolenus barathri* the data matched three species. While the BOLD identification engine (IDE) failed to provide species-level identifications for a further 11 species, in each of these cases the cause appears to be either misidentified samples on BOLD, or the limitations of distance-based methods to separate species with very shallow divergences between them, rather than the failure of DNA barcoding per se (see Discussion).

## Discussion

### Choice of BOLD database

While there are strict standards for official ‘BARCODE’ records, relating to vouchering of specimens in registered scientific collections, minimum sequence length and quality, deposition of sequence trace files, etc., BOLD also serves as a project workbench and therefore contains a mix of sequences of various lengths and with varying degrees of annotation and accuracy. Some records, particularly unpublished ones, carry interim species names. Therefore, the full BOLD database contains some inaccurately identified sequences.

The BOLD IDE uses different strategies to deal with inaccuracies in the underlying data. One strategy the IDE uses to avoid false positives is to only return an unambiguous species level identification when there is only one species name associated with the matching BOLD Barcode Index Number, or BIN (BINs are clusters of sequences, usually corresponding to species (Ratnasingham & Hebert, 2013)). However, this may lead to false negatives if some BINs contain members that have been incorrectly identified at the species level, or when the distance between closely related species is less than about 2%, the approximate threshold for distinguishing BINs. Thus, the BOLD IDE returns a conservative identification which might be improved upon by restricting the search to a subset of BOLD data. Filtered subsets of the full BOLD database exist for this purpose (see Table 2). The choice of database is a trade-off between the quantity and quality of data available for identifications.

**Table 2:** Number of animal sequences in the different BOLD databases as of November 27, 2018.

Database	No. of sequences	No. of species	No. of interim species
1. All barcode records on BOLD	5,882,500	(not stated)	(not stated)
2. Species level barcode records	3,235,340	194,552	79,026
3. Public record barcode database	1,265,200	103,980	27,962
4. Full length record barcode database	2,035,212	175,372	65,335

Our results suggest that no single BOLD database is optimal for all samples. Searches of both the Species Level Barcode Records and the Public Record Barcode Database were required to identify our samples.

### Identification of samples

It is useful to distinguish between samples which were incorrectly identified (‘Misidentified samples’) and those samples that could not be unambiguously identified at the species level. In the latter category we distinguish among samples for which there is not enough variation in the COI gene sequence to unambiguously discriminate species, vs. samples which have low levels of variation, too low to discriminate species using the methods provided on BOLD, but which can be discriminated using different analytical tools such as BLAST searches. Finally, there were those species which could not be discriminated on BOLD at this time, but this appears to be only because of the presence of misidentified sequences on BOLD, which currently obscure relationships among species. Each of these four categories is dealt with separately below.

#### Misidentified samples

DNA barcode data revealed that four samples (indicated by asterisks in Table 1) were mislabelled. Each sample is discussed below.

Blue Warehou (*Seriolella brama*) was substituted with the closely related Silver Warehou (*Spirodela punctata*). The former has a reputation as a good eating fish, however, it has been overfished and its population has not rebounded after more than a decade, according to Australia’s Sustainable Seafood Guide (Australian Marine Conservation Society, 2014). The Silver Warehou has less appeal in food markets as it is perceived as lower quality for eating, but it is an abundant species in South East Australia. Blue Warehou, on the other hand, is listed as ‘conservation dependent’ under Australian Commonwealth environmental legislation, although that still permits targeted fishing of this species (Australian Fisheries Management Authority, 2016).

A sample sold as Bream (SGS209), which is a generic term referring to members of the family Sparidae, was identified as Jackass Morwong, *Nemadactylus macropterus*, family Cheilodactylidae. This species is listed as overfished by the NSW Department of Primary Industries but as sustainable by the Australian Fisheries Management Authority (2016).

A sample of Latchet (SGS115), which is a type of Gurnard, *Pterygotrigla polyommata*, was identified as either Red Gurnard, *Chelidonichthys kumu*, or Spiny Red Gurnard, *Chelidonichthys spinosus*. These fishes look similar and this is likely a genuine case of mistaken identity rather than intentional mislabelling.

A single sample of Flathead (SGS117) was also identified as either Red Gurnard, *Chelidonichthys kumu*, or Spiny Red Gurnard, *Chelidonichthys spinosus*, although this sample was sequenced only once and cross-contamination or mislabelling of samples cannot be ruled out.

#### Species which cannot be identified to species level using DNA barcodes

Striped Marlin (*K. audax*) and White Marlin (*K. albida*) cannot be distinguished using DNA barcodes. Hanner et al. (2011) presented both COI data and data from a nuclear gene, rhodopsin, from both species with the same result, and suggested that the taxonomy of these species may need revision. Regardless, the species can be separated by incorporating information on their geographic distribution as the former species is found in the Indian and Pacific Oceans while the latter species is found in the Atlantic Ocean.

Two samples of Big-Eye Ocean Perch, *Helicolenus barathri*, and one of Ocean Perch (*Helicolenus barathri* or *Helicolenus percoides*) were identified by BOLD as one of three species, *Helicolenus barathri*, *Helicolenus lahillei*, or *Helicolenus dactylopterus*. It is unclear whether DNA barcodes lack the resolving power to distinguish these species or whether there are some misidentified specimens on BOLD. However, we note that the latter two species occur in the Atlantic Ocean while *Helicolenus barathri* occurs in the Southwest Pacific, so they may be distinguished by their place of origin, if that information is available. Furthermore, the other Southwest Pacific species, *Helicolenus percoides*, is easily distinguished from the other three species by DNA barcodes.

Two samples of Blue Grenadier, *M. novaezelandiae*, and two samples of Hoki, either *M. novaezelandiae* or *M. magellanicus*, were identified by BOLD IDE as either of these two species, that is, DNA barcode data cannot distinguish the two species. However, the online Catalog of Fishes (Eschmeyer et al., 2018) lists *M. magellanicus* as a junior synonym of *M. novaezelandiae*.

For Sand Whiting, *Sillago ciliata*, BOLD gave 100% matches to two species, *Sillago ciliata* and *Stenoria analis*. This lack of resolution was investigated more thoroughly by Krück et al. (2013) using both mitochondrial and nuclear genes. The authors found that a single site in the DNA barcode region of COI was usually diagnostic for these two species, however, that was not the case for three of their 60 samples (5%), which showed discordance between nuclear and mitochondrial gene phylogenies, evidence of a past hybridization event. Nuclear gene data are therefore required to distinguish these two species. The ranges of these two commercial species overlap in Queensland so it may prove important to distinguish these species in the Queensland fishery.

#### Samples not identified to species level by BOLD IDE, but identifiable with other methods

Tuna (*Thunnus*) species cannot be accurately identified by the BOLD IDE because it relies on threshold distances and neighbour-joining trees, and there is insufficient COI sequence divergence between species for these methods to accurately distinguish species. However, Lowenstein, Amato & Kolokotronis (2009) demonstrated that DNA barcode data can be used to distinguish *Thunnus* species if one uses a character-based approach or if one simply takes the closest match in a BLAST search (or on BOLD). We used the latter approach to refine our identification of tuna samples, and concluded that both were correctly identified as Yellowfin Tuna, *Thunnus albacares*.

Similarly, the BOLD IDE identified our two samples of Yellowtail Scad, *Trachurus novaezelandiae*, as one of three species, *Trachurus novaezelandiae*, *Trachurus declivis*, or *Trissolcus japonicus*, however the closest matches were to *Trachurus novaezelandiae*. Furthermore, using the BOLD Published database, NJ trees showed clear separation of *Trachurus novaezelandiae* from the other two species.

Eastern Sea Garfish, *Hyporhamphus australis*, shows a similar pattern on BOLD, with very low divergences among three species, however a BOLD NJ tree separates *Hyporhamphus australis* from the other *Hyporhamphus* species, *Hyporhamphus melanochir* and *Hyporhamphus ihi*.

#### Samples not identified to species level by BOLD IDE, possibly due to misidentified reference samples on BOLD

For *Hyporhamphus australis*, a sequence labelled as *Arrhamphus sclerolepis* is also clustered with *Hyporhamphus melanochir* sequences. However, that *A. sclerolepis* sequence is well separated in the NJ tree from conspecific sequences and would appear to be misidentified.

Goldband Snapper, *Pristipomoides multidens*, was identified by BOLD IDE as either *Pristipomoides multidens* or *Pomadasys maculatus*. Yet, the single matching sample of *Pomadasys maculatus* has clearly been misidentified as it belongs to a different family and the other >50 representatives of this species on BOLD are distantly related and are not found in BOLD searches using *Pristipomoides multidens* sequences.

Yellowtail Kingfish, *Seriola lalandi*, was identified by BOLD IDE as *Seriola lalandi* or *Schistura zonata*. BOLD IDE NJ trees show *Seriola lalandi* as a single cluster of some 30 samples, with five samples of *Schistura zonata* from Brazil nested within this cluster. There is <1% distance among members of this cluster. However, there is a second cluster of *Schistura zonata* samples from the USA, placed >7% distant from *Seriola lalandi*, which suggests that the Brazil samples may have been misidentified (or the taxonomy is incorrect). In this case the Brazil ‘*Schistura zonata*’ sequences are in the Public Record Database while the USA sequences are unpublished.

For *Centroberyx affinis*, a search of the BOLD Public Record Database provided a 98.3% match to a single specimen of this species. However, searching the Species Level Record Database found a 100% match to a cluster of unpublished sequences of both *Centroberyx affinis* and *Centroberyx australis*. Supposed *C. australis* sequences group into three different barcode clusters. Thus, our sample is likely correctly identified, but no firm conclusions can be drawn at this stage.

Our single Shark sample was identified as *Carcharhinus brevipinna*. A single record of *Carcharhinus limbatus* was embedded within the cluster of *Carcharhinus brevipinna* on BOLD. That single record is well separated from the many other *Carcharhinus limbatus* on BOLD and thus likely represents a misidentified specimen. This is an example of the limitations of the BOLD database and highlights the importance of database validation and utility of museum voucher collections.

Finally, the Bluespotted Goatfish, *Upeneichthys vlamingii*, was identified by BOLD IDE using the Species Level Record Database as either *Upeneichthys vlamingii* or *Uroplectes lineatus*. However, the two *Uroplectes lineatus* records on BOLD are both unpublished and are separated by >4.5% COI distance, so it appears likely that one of the *Uroplectes lineatus* sequences has been misidentified.

### Utility of DNA barcode data for fish species authentication

Using DNA barcode data we could identify 35 of 39 species sampled (90%) to species level and all samples to genus level. However, the identification tools on BOLD provided unambiguous identifications for only 27 samples (69%) due to the presence of potentially misidentified records on BOLD, poorly resolved taxonomy and/or the limitations of the BOLD IDE. There were eight species (21%) which required further investigation, which entailed reading the published scientific literature on DNA barcoding of fishes to assess the validity of identifications attached to published COI sequences, and applying alternative methods (e.g. NCBI BLAST searches) to gain identifications.

DNA databases used for identification of regulated species require high levels of data integrity and redundancy (Frewin, Scott-Dupree & Hanner, 2013; Mitchell & Gopurenko, 2016). The putative identification errors on BOLD mentioned above illustrate the problems inherent in having a database serve as both a workbench for projects in progress and a reference database. One possible approach to dealing with this issue is to separate these functions by creating a separate ‘reference database’ as an additional subset of BOLD sequences, which can be selected when identifying regulated species. The US FDA’s designation of reference sequences for some species of fish is a step in that direction (Handy et al., 2011). BOLD is already a valuable platform for identifying fish products but further research and development is needed to ensure the accuracy and future utility of BOLD data for regulatory purposes in Australia.

### Labelling of fish products

#### Use of standard names

The AFNS AS SSA 5300-2015 (AFNS) specifies that fish sold to consumers (i.e. retail sales and restaurants) must be identified by their standard fish name. However, the AFNS is a voluntary standard and Food Standards Australia and New Zealand (FSANZ) does not mandate compliance with the standard (Senate Standing Committee on Rural and Regional Affairs and Transport, 2014). A total of 20 species of fish that we sampled, or 40% of our sample, did not precisely comply with the standard. However, if we exclude spelling errors and cases where vendors supplied more information than the standard requires (e.g. Rainbow Trout rather than just Trout) then the error rate drops to 22%, which is still substantial. Many published studies have not differentiated between mislabelling due to the use of unofficial names, and actual misidentification or misrepresentation of species.

#### Accuracy of product labels

DNA barcodes confirmed the retailers’ identifications of all but four of the 61 samples and 39 species sampled, that is, 7% of samples and 10% of species were mislabelled. While this is poorer than the only other published market survey in Australia that used DNA barcodes (Lamendin, Miller & Ward, 2015) it is at the lower end of the range of mislabelling rates reported from surveys conducted in other countries. In a review of 51 seafood authentication studies Pardo, Jiménez & Pérez-Villarreal (2016) reported that the average rate of mislabelling was 30%. Restaurants and takeaways generally recorded higher levels of mislabelling than fishmongers and supermarkets, but only comprised 10% of samples analysed in all studies. Our survey was restricted to two major retailers of fresh fish in the Sydney central business district, selling mostly Australian produced fish. It is possible that different results would be obtained if sampling imported fish, or other localities in Australia, or restaurants.

#### Adequacy of the Australian fish names standard

As a matter of principle, wild caught IUCN Red-Listed species should not be sold for food. At the very least, consumers should be empowered to make choices that support sustainable use of natural resources. This is impossible when retailers are permitted to group many species under one label, for example, ‘Snapper’ is an umbrella term that has found to be applied to more than 60 species in 16 fish families (Cawthorn, Baillie & Mariani, 2018). An example in the AFNS is Basa. Our single sample of Basa was identified as *Pangasianodon hypophthalmus*. The AFNS defines Basa as any member of the family Pangasiidae, therefore this name is compliant. However, the AFNS usage of this name is extremely broad, and is inconsistent with global standards. On the United Nations Food and Agriculture Organization fish names list, *Pangasianodon hypophthalmus* is called Swai, while Basa is reserved for *Pangasius bocourti*. Both species are farmed in Asia, but natural populations of *Pangasianodon hypophthalmus* are Red-listed as Endangered. Two other Pangasiidae, *Pangasianodon gigas* and *Pangasius sanitwongsei*, are Red-listed as Critically Endangered. DNA barcode data provides robust discrimination of these species, yet any of them can be called Basa in Australia. Ideally, the names standard would distinguish between these species and also between wild and farmed populations of these species. Furthermore, the industry should only allow imports of species in the Least Concern category.

### DNA barcoding in the classroom

Evidence suggests that being actively involved in original research improves student engagement and retention (Henter et al., 2016). Naaum & Hanner (2015) reported results of a project similar in scope and have also made teaching resources available online for educators. The BOLD SDP (Santschi et al., 2013) facilitates direct participation in DNA barcoding research by citizen scientists of all capabilities, with simple point-and-click tools for data entry and analysis as well as check-points for data quality control managed by course instructors and participating scientists, before data are submitted to online databases. In this study, the bulk of the laboratory and analytical work was performed by high school students who had volunteered to partake in science extension courses. However, if the learning experiences of students can be enhanced through more active learning processes, such as that employed here (and elsewhere) there is scope for incorporating DNA barcoding research into curricula at both secondary (Henter et al., 2016) and tertiary (Borrell et al., 2016) levels.

In an educational context, this project generates greater awareness of the issue of sustainability and provides students with an opportunity to be involved in current and topical research. At the secondary level, the project has a relevance and pertinence to the implementation of a new curriculum in Australia, which promotes student learning in connection with the reliance of Biology in their lives, along with the science extension curriculum which encourages students to work with real scientific datasets. For example, it links with topics on Future Ecosystems with inquiry questions including ‘how can human activity impact on an ecosystem’ and on sustainability and awareness in the community, which provides opportunities to reflect on food sources and human interaction with their environment. It also aims to build recognition of the influence of advancing scientific knowledge contributing towards economic, political, and social facets in the community. The project lends itself well to the Depth Studies, which have a research component, allowing students a chance to further develop their interest in biology and take ownership of their learning.

Undertaking research increases the motivation of students (Franzoni & Sauermann, 2014) and marries perfectly with the NSW Education Standards Authority (2017) ideals of students taking responsibility for their own learning. Citizen science projects are increasingly demonstrating improved learning and interest (Freeman et al., 2014). This project provided students with a real-life experience of the importance of science and enabled them to engage with it directly as well as interact directly with practicing research scientists, rather than just theorise on or learn about due to mandated syllabus guidelines in the classroom. Such projects truly allow the development of young scientific minds with a cognizant, up to date knowledge and positive attitudes towards biology.

## Conclusions

High school students performed the bulk of the laboratory and analytical work for this study, under the guidance of teachers and scientists, demonstrating that this can be an effective way both to teach students about biodiversity, genetics, sustainability, and other environmental issues, and to gather and analyse scientific data.

DNA barcoding is proving to be an enormously useful tool for identifying fish species, whether the ultimate purpose is seafood authentication, fisheries management, or taxonomic research. However, we note that much further research and development is needed to maximize the scope and reliability of barcode data stored in the online BOLD database.

The past few years have seen a surge in public interest in seafood labelling standards in Australia, as witnessed by increasing media coverage. FSANZ is the statutory authority responsible for developing food standards in Australia and New Zealand. When developing food standards, FSANZ’s primary objectives are the protection of public health and safety, the provision of adequate information to enable consumers to make informed choices, and the prevention of misleading conduct (Senate Standing Committee on Rural and Regional Affairs and Transport, 2014). Market surveys such as the current study are useful for assessing the degree to which these objectives are being met by current legislation. We found at best 78% and at worst only 60% of samples complied with the AFNS in our small sample of Sydney’s fresh fish retailers. In addition, 7% of samples were misidentified. Further studies are needed to get a more reliable estimate of compliance levels both in Sydney and in other regions, in different markets, for example, for restaurants vs. fresh fish retailers, for imported vs. Australian produced fish products and for different seafood products, in order to inform policy in this area.

## Supplemental Information

10.7717/peerj.7138/supp-1 Supplemental Information 1 BOLD tree identification–sample SGS224–Thunnus albacares.

10.7717/peerj.7138/supp-2 Supplemental Information 2 BOLD tree identification–sample–sample SGS233-Hyporhamphus australis.

10.7717/peerj.7138/supp-3 Supplemental Information 3 BOLD ID engine–sample SGS233-Hyporhamphus australis.

10.7717/peerj.7138/supp-4 Supplemental Information 4 BOLD ID engine–sample SGS224–Thunnus albacares.

## Additional Information and Declarations

### DNA Deposition

The following information was supplied regarding the deposition of DNA sequences: The sequences are available at GenBank: KX781849–KX781869, KX781872–KX781885, KX781889–KX781892, KX781921–KX781925, KX781931–KX781946.

### Data Availability

The following information was supplied regarding data availability: Sequence trace files and sample photographs are available on BOLD: http://www.boldsystems.org/index.php/Public_SearchTerms?query=SDP331.
