# Supplementary material for: Could do better! A high school market survey of fish labelling in Sydney, Australia, using DNA barcodes
Source: PeerJ. 2019 Jun 14;7:e7138. doi: 10.7717/peerj.7138 (PMC6573807; doi:10.7717/peerj.7138)
Supplement: Supplemental Information 1 [file peerj-07-7138-s001.pdf]

BOLD TaxonID Tree

Title	: COI SPECIES DATABASE Tree
Date	: 27-March-2019
Data Type	: Nucleotide
Distance Model	: Kimura 2 Parameter
Marker	: COI-5P
Codon Positions	: 1st, 2nd, 3rd
Labels	: Extra Info, Country & Province, Family
Filters	: Length > 200
Attachment	: Photographs & Spreadsheet

Sequence Count	: 100
Species count	: 3
Genus count	: 1
Family count	: 1
Unidentified	: 1

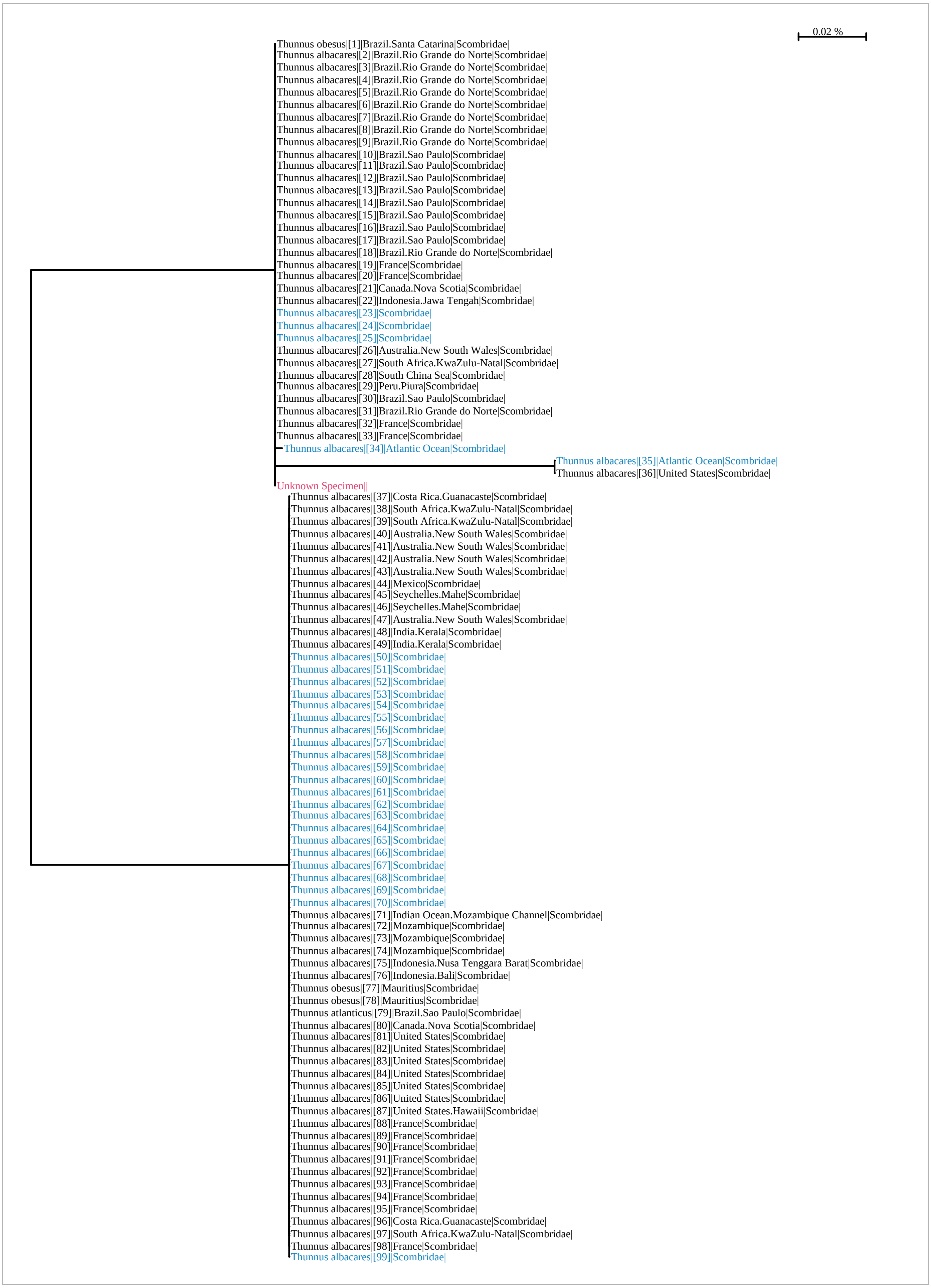
0.02 %
Thunnus obesus|[1]|Brazil.Santa Catarina|Scombridae|
Thunnus albacares|[2]|Brazil.Rio Grande do Norte|Scombridae|
Thunnus albacares|[3]|Brazil.Rio Grande do Norte|Scombridae|
Thunnus albacares|[4]|Brazil.Rio Grande do Norte|Scombridae|
Thunnus albacares|[5]|Brazil.Rio Grande do Norte|Scombridae|
Thunnus albacares|[6]|Brazil.Rio Grande do Norte|Scombridae|
Thunnus albacares|[7]|Brazil.Rio Grande do Norte|Scombridae|
Thunnus albacares|[8]|Brazil.Rio Grande do Norte|Scombridae|
Thunnus albacares|[9]|Brazil.Rio Grande do Norte|Scombridae|
Thunnus albacares|[10]|Brazil.Sao Paulo|Scombridae|
Thunnus albacares|[11]|Brazil.Sao Paulo|Scombridae|
Thunnus albacares|[12]|Brazil.Sao Paulo|Scombridae|
Thunnus albacares|[13]|Brazil.Sao Paulo|Scombridae|
Thunnus albacares|[14]|Brazil.Sao Paulo|Scombridae|
Thunnus albacares|[15]|Brazil.Sao Paulo|Scombridae|
Thunnus albacares|[16]|Brazil.Sao Paulo|Scombridae|
Thunnus albacares|[17]|Brazil.Sao Paulo|Scombridae|
Thunnus albacares|[18]|Brazil.Rio Grande do Norte|Scombridae|
Thunnus albacares|[19]|France|Scombridae|
Thunnus albacares|[20]|France|Scombridae|
Thunnus albacares|[21]|Canada.Nova Scotia|Scombridae|
Thunnus albacares|[22]|Indonesia.Jawa Tengah|Scombridae|
Thunnus albacares|[23]|Scombridae|
Thunnus albacares|[24]|Scombridae|
Thunnus albacares|[25]|Scombridae|
Thunnus albacares|[26]|Australia.New South Wales|Scombridae|
Thunnus albacares|[27]|South Africa.KwaZulu-Natal|Scombridae|
Thunnus albacares|[28]|South China Sea|Scombridae|
Thunnus albacares|[29]|Peru.Piura|Scombridae|
Thunnus albacares|[30]|Brazil.Sao Paulo|Scombridae|
Thunnus albacares|[31]|Brazil.Rio Grande do Norte|Scombridae|
Thunnus albacares|[32]|France|Scombridae|
Thunnus albacares|[33]|France|Scombridae|
Thunnus albacares|[34]|Atlantic Ocean|Scombridae|
Thunnus albacares|[35]|Atlantic Ocean|Scombridae|
Thunnus albacares|[36]|United States|Scombridae|
Unknown Specimen||
Thunnus albacares|[37]|Costa Rica.Guanacaste|Scombridae|
Thunnus albacares|[38]|South Africa.KwaZulu-Natal|Scombridae|
Thunnus albacares|[39]|South Africa.KwaZulu-Natal|Scombridae|
Thunnus albacares|[40]|Australia.New South Wales|Scombridae|
Thunnus albacares|[41]|Australia.New South Wales|Scombridae|
Thunnus albacares|[42]|Australia.New South Wales|Scombridae|
Thunnus albacares|[43]|Australia.New South Wales|Scombridae|
Thunnus albacares|[44]|Mexico|Scombridae|
Thunnus albacares|[45]|Seychelles.Mahe|Scombridae|
Thunnus albacares|[46]|Seychelles.Mahe|Scombridae|
Thunnus albacares|[47]|Australia.New South Wales|Scombridae|
Thunnus albacares|[48]|India.Kerala|Scombridae|
Thunnus albacares|[49]|India.Kerala|Scombridae|
Thunnus albacares|[50]|Scombridae|
Thunnus albacares|[51]|Scombridae|
Thunnus albacares|[52]|Scombridae|
Thunnus albacares|[53]|Scombridae|
Thunnus albacares|[54]|Scombridae|
Thunnus albacares|[55]|Scombridae|
Thunnus albacares|[56]|Scombridae|
Thunnus albacares|[57]|Scombridae|
Thunnus albacares|[58]|Scombridae|
Thunnus albacares|[59]|Scombridae|
Thunnus albacares|[60]|Scombridae|
Thunnus albacares|[61]|Scombridae|
Thunnus albacares|[62]|Scombridae|
Thunnus albacares|[63]|Scombridae|
Thunnus albacares|[64]|Scombridae|
Thunnus albacares|[65]|Scombridae|
Thunnus albacares|[66]|Scombridae|
Thunnus albacares|[67]|Scombridae|
Thunnus albacares|[68]|Scombridae|
Thunnus albacares|[69]|Scombridae|
Thunnus albacares|[70]|Scombridae|
Thunnus albacares|[71]|Indian Ocean.Mozambique Channel|Scombridae|
Thunnus albacares|[72]|Mozambique|Scombridae|
Thunnus albacares|[73]|Mozambique|Scombridae|
Thunnus albacares|[74]|Mozambique|Scombridae|
Thunnus albacares|[75]|Indonesia.Nusa Tenggara Barat|Scombridae|
Thunnus albacares|[76]|Indonesia.Bali|Scombridae|
Thunnus obesus|[77]|Mauritius|Scombridae|
Thunnus obesus|[78]|Mauritius|Scombridae|
Thunnus atlanticus|[79]|Brazil.Sao Paulo|Scombridae|
Thunnus albacares|[80]|Canada.Nova Scotia|Scombridae|
Thunnus albacares|[81]|United States|Scombridae|
Thunnus albacares|[82]|United States|Scombridae|
Thunnus albacares|[83]|United States|Scombridae|
Thunnus albacares|[84]|United States|Scombridae|
Thunnus albacares|[85]|United States|Scombridae|
Thunnus albacares|[86]|United States|Scombridae|
Thunnus albacares|[87]|United States.Hawaii|Scombridae|
Thunnus albacares|[88]|France|Scombridae|
Thunnus albacares|[89]|France|Scombridae|
Thunnus albacares|[90]|France|Scombridae|
Thunnus albacares|[91]|France|Scombridae|
Thunnus albacares|[92]|France|Scombridae|
Thunnus albacares|[93]|France|Scombridae|
Thunnus albacares|[94]|France|Scombridae|
Thunnus albacares|[95]|France|Scombridae|
Thunnus albacares|[96]|Costa Rica.Guanacaste|Scombridae|
Thunnus albacares|[97]|South Africa.KwaZulu-Natal|Scombridae|
Thunnus albacares|[98]|France|Scombridae|
Thunnus albacares|[99]|Scombridae|
