# Supplementary material for: Could do better! A high school market survey of fish labelling in Sydney, Australia, using DNA barcodes
Source: PeerJ. 2019 Jun 14;7:e7138. doi: 10.7717/peerj.7138 (PMC6573807; doi:10.7717/peerj.7138)
Supplement: Supplemental Information 2 [file peerj-07-7138-s002.pdf]

BOLD TaxonID Tree

Title	:	COI SPECIES DATABASE Tree
Date	:	27-March-2019
Data Type	:	Nucleotide
Distance Model	:	Kimura 2 Parameter
Marker	:	COI-5P
Codon Positions	:	1st, 2nd, 3rd
Labels	:	Extra Info, Country & Province, Family
Filters	:	Length > 200
Attachment	:	Photographs & Spreadsheet

Sequence Count	:	100
Species count	:	19
Genus count	:	4
Family count	:	1
Unidentified	:	1

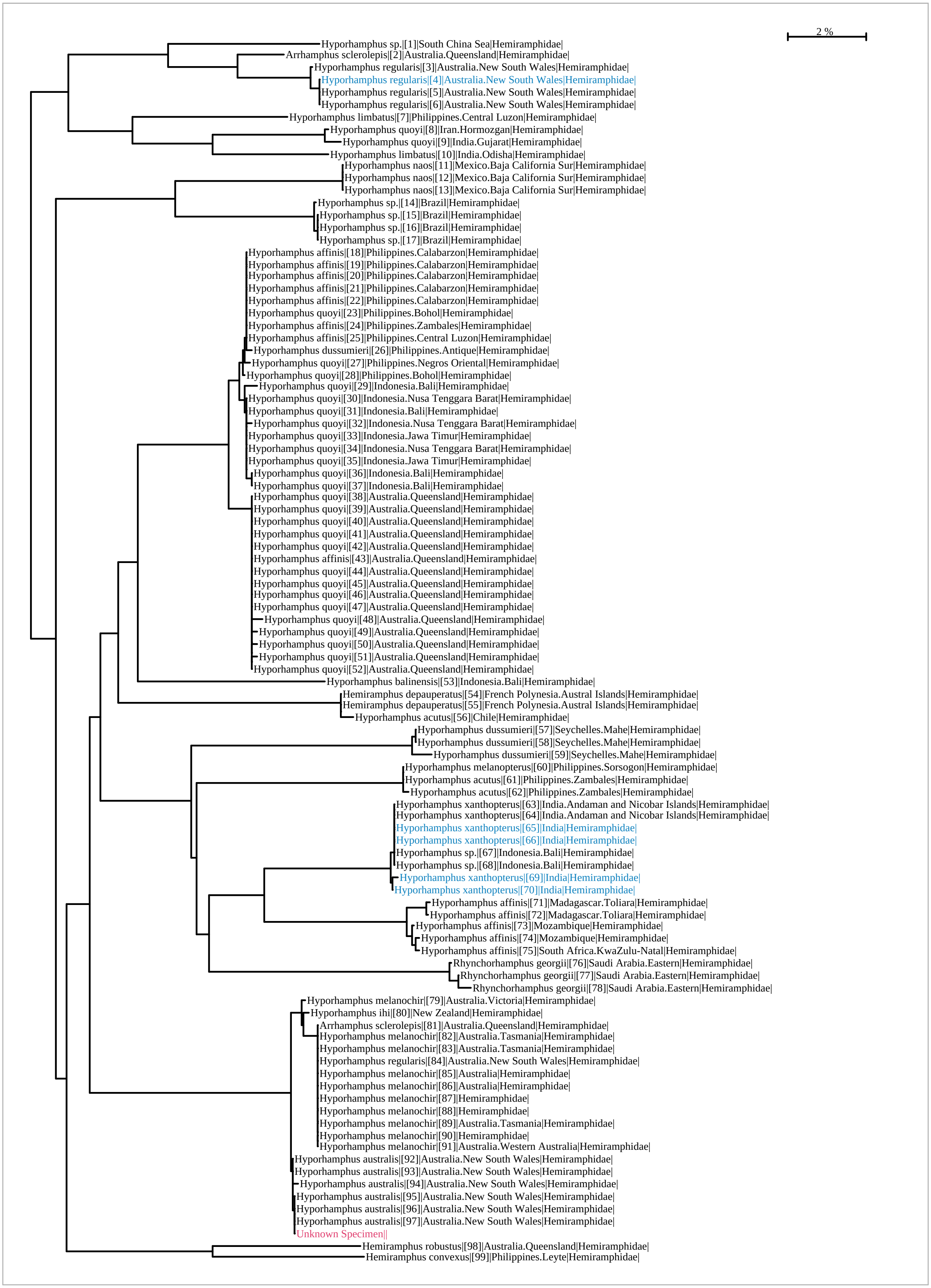
2 %
Hyporhamphus sp.|[1]|South China Sea|Hemiramphidae|
Arrhamphus sclerolepis|[2]|Australia.Queensland|Hemiramphidae|
Hyporhamphus regularis|[3]|Australia.New South Wales|Hemiramphidae|
Hyporhamphus regularis|[4]|Australia.New South Wales|Hemiramphidae|
Hyporhamphus regularis|[5]|Australia.New South Wales|Hemiramphidae|
Hyporhamphus regularis|[6]|Australia.New South Wales|Hemiramphidae|
Hyporhamphus limbatus|[7]|Philippines.Central Luzon|Hemiramphidae|
Hyporhamphus quoyi|[8]|Iran.Hormozgan|Hemiramphidae|
Hyporhamphus quoyi|[9]|India.Gujarat|Hemiramphidae|
Hyporhamphus limbatus|[10]|India.Odisha|Hemiramphidae|
Hyporhamphus naos|[11]|Mexico.Baja California Sur|Hemiramphidae|
Hyporhamphus naos|[12]|Mexico.Baja California Sur|Hemiramphidae|
Hyporhamphus naos|[13]|Mexico.Baja California Sur|Hemiramphidae|
Hyporhamphus sp.|[14]|Brazil|Hemiramphidae|
Hyporhamphus sp.|[15]|Brazil|Hemiramphidae|
Hyporhamphus sp.|[16]|Brazil|Hemiramphidae|
Hyporhamphus sp.|[17]|Brazil|Hemiramphidae|
Hyporhamphus affinis|[18]|Philippines.Calabarzon|Hemiramphidae|
Hyporhamphus affinis|[19]|Philippines.Calabarzon|Hemiramphidae|
Hyporhamphus affinis|[20]|Philippines.Calabarzon|Hemiramphidae|
Hyporhamphus affinis|[21]|Philippines.Calabarzon|Hemiramphidae|
Hyporhamphus affinis|[22]|Philippines.Calabarzon|Hemiramphidae|
Hyporhamphus quoyi|[23]|Philippines.Bohol|Hemiramphidae|
Hyporhamphus affinis|[24]|Philippines.Zambales|Hemiramphidae|
Hyporhamphus affinis|[25]|Philippines.Central Luzon|Hemiramphidae|
Hyporhamphus dussumieri|[26]|Philippines.Antique|Hemiramphidae|
Hyporhamphus quoyi|[27]|Philippines.Negros Oriental|Hemiramphidae|
Hyporhamphus quoyi|[28]|Philippines.Bohol|Hemiramphidae|
Hyporhamphus quoyi|[29]|Indonesia.Bali|Hemiramphidae|
Hyporhamphus quoyi|[30]|Indonesia.Nusa Tenggara Barat|Hemiramphidae|
Hyporhamphus quoyi|[31]|Indonesia.Bali|Hemiramphidae|
Hyporhamphus quoyi|[32]|Indonesia.Nusa Tenggara Barat|Hemiramphidae|
Hyporhamphus quoyi|[33]|Indonesia.Jawa Timur|Hemiramphidae|
Hyporhamphus quoyi|[34]|Indonesia.Nusa Tenggara Barat|Hemiramphidae|
Hyporhamphus quoyi|[35]|Indonesia.Jawa Timur|Hemiramphidae|
Hyporhamphus quoyi|[36]|Indonesia.Bali|Hemiramphidae|
Hyporhamphus quoyi|[37]|Indonesia.Bali|Hemiramphidae|
Hyporhamphus quoyi|[38]|Australia.Queensland|Hemiramphidae|
Hyporhamphus quoyi|[39]|Australia.Queensland|Hemiramphidae|
Hyporhamphus quoyi|[40]|Australia.Queensland|Hemiramphidae|
Hyporhamphus quoyi|[41]|Australia.Queensland|Hemiramphidae|
Hyporhamphus quoyi|[42]|Australia.Queensland|Hemiramphidae|
Hyporhamphus affinis|[43]|Australia.Queensland|Hemiramphidae|
Hyporhamphus quoyi|[44]|Australia.Queensland|Hemiramphidae|
Hyporhamphus quoyi|[45]|Australia.Queensland|Hemiramphidae|
Hyporhamphus quoyi|[46]|Australia.Queensland|Hemiramphidae|
Hyporhamphus quoyi|[47]|Australia.Queensland|Hemiramphidae|
Hyporhamphus quoyi|[48]|Australia.Queensland|Hemiramphidae|
Hyporhamphus quoyi|[49]|Australia.Queensland|Hemiramphidae|
Hyporhamphus quoyi|[50]|Australia.Queensland|Hemiramphidae|
Hyporhamphus quoyi|[51]|Australia.Queensland|Hemiramphidae|
Hyporhamphus quoyi|[52]|Australia.Queensland|Hemiramphidae|
Hyporhamphus balinensis|[53]|Indonesia.Bali|Hemiramphidae|
Hemiramphus depauperatus|[54]|French Polynesia.Austral Islands|Hemiramphidae|
Hemiramphus depauperatus|[55]|French Polynesia.Austral Islands|Hemiramphidae|
Hyporhamphus acutus|[56]|Chile|Hemiramphidae|
Hyporhamphus dussumieri|[57]|Seychelles.Mahe|Hemiramphidae|
Hyporhamphus dussumieri|[58]|Seychelles.Mahe|Hemiramphidae|
Hyporhamphus dussumieri|[59]|Seychelles.Mahe|Hemiramphidae|
Hyporhamphus melanopterus|[60]|Philippines.Sorsogon|Hemiramphidae|
Hyporhamphus acutus|[61]|Philippines.Zambales|Hemiramphidae|
Hyporhamphus acutus|[62]|Philippines.Zambales|Hemiramphidae|
Hyporhamphus xanthopterus|[63]|India.Andaman and Nicobar Islands|Hemiramphidae|
Hyporhamphus xanthopterus|[64]|India.Andaman and Nicobar Islands|Hemiramphidae|
Hyporhamphus xanthopterus|[65]|India|Hemiramphidae|
Hyporhamphus xanthopterus|[66]|India|Hemiramphidae|
Hyporhamphus sp.|[67]|Indonesia.Bali|Hemiramphidae|
Hyporhamphus sp.|[68]|Indonesia.Bali|Hemiramphidae|
Hyporhamphus xanthopterus|[69]|India|Hemiramphidae|
Hyporhamphus xanthopterus|[70]|India|Hemiramphidae|
Hyporhamphus affinis|[71]|Madagascar.Toliara|Hemiramphidae|
Hyporhamphus affinis|[72]|Madagascar.Toliara|Hemiramphidae|
Hyporhamphus affinis|[73]|Mozambique|Hemiramphidae|
Hyporhamphus affinis|[74]|Mozambique|Hemiramphidae|
Hyporhamphus affinis|[75]|South Africa.KwaZulu-Natal|Hemiramphidae|
Rhynchorhamphus georgii|[76]|Saudi Arabia.Eastern|Hemiramphidae|
Rhynchorhamphus georgii|[77]|Saudi Arabia.Eastern|Hemiramphidae|
Rhynchorhamphus georgii|[78]|Saudi Arabia.Eastern|Hemiramphidae|
Hyporhamphus melanochir|[79]|Australia.Victoria|Hemiramphidae|
Hyporhamphus ihi|[80]|New Zealand|Hemiramphidae|
Arrhamphus sclerolepis|[81]|Australia.Queensland|Hemiramphidae|
Hyporhamphus melanochir|[82]|Australia.Tasmania|Hemiramphidae|
Hyporhamphus melanochir|[83]|Australia.Tasmania|Hemiramphidae|
Hyporhamphus regularis|[84]|Australia.New South Wales|Hemiramphidae|
Hyporhamphus melanochir|[85]|Australia|Hemiramphidae|
Hyporhamphus melanochir|[86]|Australia|Hemiramphidae|
Hyporhamphus melanochir|[87]|Hemiramphidae|
Hyporhamphus melanochir|[88]|Hemiramphidae|
Hyporhamphus melanochir|[89]|Australia.Tasmania|Hemiramphidae|
Hyporhamphus melanochir|[90]|Hemiramphidae|
Hyporhamphus melanochir|[91]|Australia.Western Australia|Hemiramphidae|
Hyporhamphus australis|[92]|Australia.New South Wales|Hemiramphidae|
Hyporhamphus australis|[93]|Australia.New South Wales|Hemiramphidae|
Hyporhamphus australis|[94]|Australia.New South Wales|Hemiramphidae|
Hyporhamphus australis|[95]|Australia.New South Wales|Hemiramphidae|
Hyporhamphus australis|[96]|Australia.New South Wales|Hemiramphidae|
Hyporhamphus australis|[97]|Australia.New South Wales|Hemiramphidae|
Unknown Specimen||
Hemiramphus robustus|[98]|Australia.Queensland|Hemiramphidae|
Hemiramphus convexus|[99]|Philippines.Leyte|Hemiramphidae|
