# Supplementary material for: Could do better! A high school market survey of fish labelling in Sydney, Australia, using DNA barcodes
Source: PeerJ. 2019 Jun 14;7:e7138. doi: 10.7717/peerj.7138 (PMC6573807; doi:10.7717/peerj.7138)
Supplement: Supplemental Information 3 [file peerj-07-7138-s003.pdf]

IDENTIFICATION ENGINE: RESULTS

Results Summary

Query ID	Best ID	Search DB	Tree	Top %	Graph	Low %
SDP331088-16|KX781932|SGS233_2016|Hyporhamphus australis|COI-5P	*Hyporhamphus australis*	COI SPECIES DATABASE		100.00	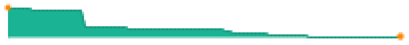	87.56

Query: SDP331088-16|KX781932|SGS233_2016|Hyporhamphus australis|COI-5P
Top Hit: Chordata Actinopterygii - Beloniformes - *Hyporhamphus australis* (100%)

Search Result:

A species level match could not be made, the queried specimen is likely to be one of the following:

Hyporhamphus australis
Hyporhamphus ihi
Hyporhamphus melanochir
Hyporhamphus regularis
Arrhamphus sclerolepis

For a hierarchical placement - a neighbor-joining tree is provided:

TREE BASED IDENTIFICATION

Identification Summary

Taxonomic Level	Taxon Assignment	Probability of Placement (%)
Phylum	Chordata	100
Class	Actinopterygii	100
Order	Beloniformes	100
Family	Hemiramphidae	100
Genus	*Hyporhamphus*	100

Similarity Scores of Top 99 Matches

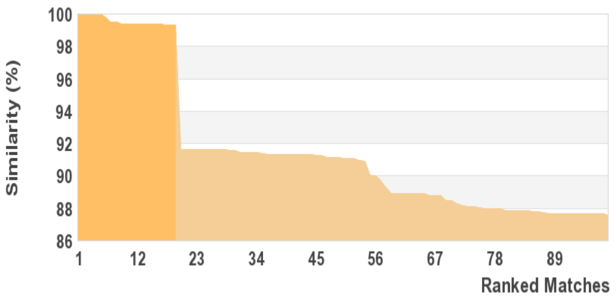

Display:

Top 20 ▼

Top 20 Matches

Phylum	Class	Order	Family	Genus	Species	Subspecies	Similarity (%)	S
Chordata	Actinopterygii	Beloniformes	Hemiramphidae	*Hyporhamphus*	*australis*		100	F
Chordata	Actinopterygii	Beloniformes	Hemiramphidae	*Hyporhamphus*	*australis*		100	F
Chordata	Actinopterygii	Beloniformes	Hemiramphidae	*Hyporhamphus*	*australis*		100	F
Chordata	Actinopterygii	Beloniformes	Hemiramphidae	*Hyporhamphus*	*australis*		100	F
Chordata	Actinopterygii	Beloniformes	Hemiramphidae	*Hyporhamphus*	*australis*		100	E F
Chordata	Actinopterygii	Beloniformes	Hemiramphidae	*Hyporhamphus*	*australis*		99.84	F

Chordata	Actinopterygii	Beloniformes	Hemiramphidae	*Hyporhamphus*	*ihi*	99.53	F
Chordata	Actinopterygii	Beloniformes	Hemiramphidae	*Hyporhamphus*	*melanochir*	99.53	F
Chordata	Actinopterygii	Beloniformes	Hemiramphidae	*Hyporhamphus*	*melanochir*	99.37	F
Chordata	Actinopterygii	Beloniformes	Hemiramphidae	*Hyporhamphus*	*melanochir*	99.37	F
Chordata	Actinopterygii	Beloniformes	Hemiramphidae	*Hyporhamphus*	*melanochir*	99.37	F
Chordata	Actinopterygii	Beloniformes	Hemiramphidae	*Hyporhamphus*	*melanochir*	99.37	F
Chordata	Actinopterygii	Beloniformes	Hemiramphidae	*Hyporhamphus*	*regularis*	99.37	E F
Chordata	Actinopterygii	Beloniformes	Hemiramphidae	*Hyporhamphus*	*melanochir*	99.37	F
Chordata	Actinopterygii	Beloniformes	Hemiramphidae	*Hyporhamphus*	*melanochir*	99.37	F
Chordata	Actinopterygii	Beloniformes	Hemiramphidae	*Arrhamphus*	*sclerolepis*	99.37	F
Chordata	Actinopterygii	Beloniformes	Hemiramphidae	*Hyporhamphus*	*melanochir*	99.35	F
Chordata	Actinopterygii	Beloniformes	Hemiramphidae	*Hyporhamphus*	*melanochir*	99.34	F
Chordata	Actinopterygii	Beloniformes	Hemiramphidae	*Hyporhamphus*	*melanochir*	99.33	F
Chordata	Actinopterygii	Beloniformes	Hemiramphidae	*Hyporhamphus*	*quoyi*	91.63	F

Sampling Sites For Top Hits (>98% Match)

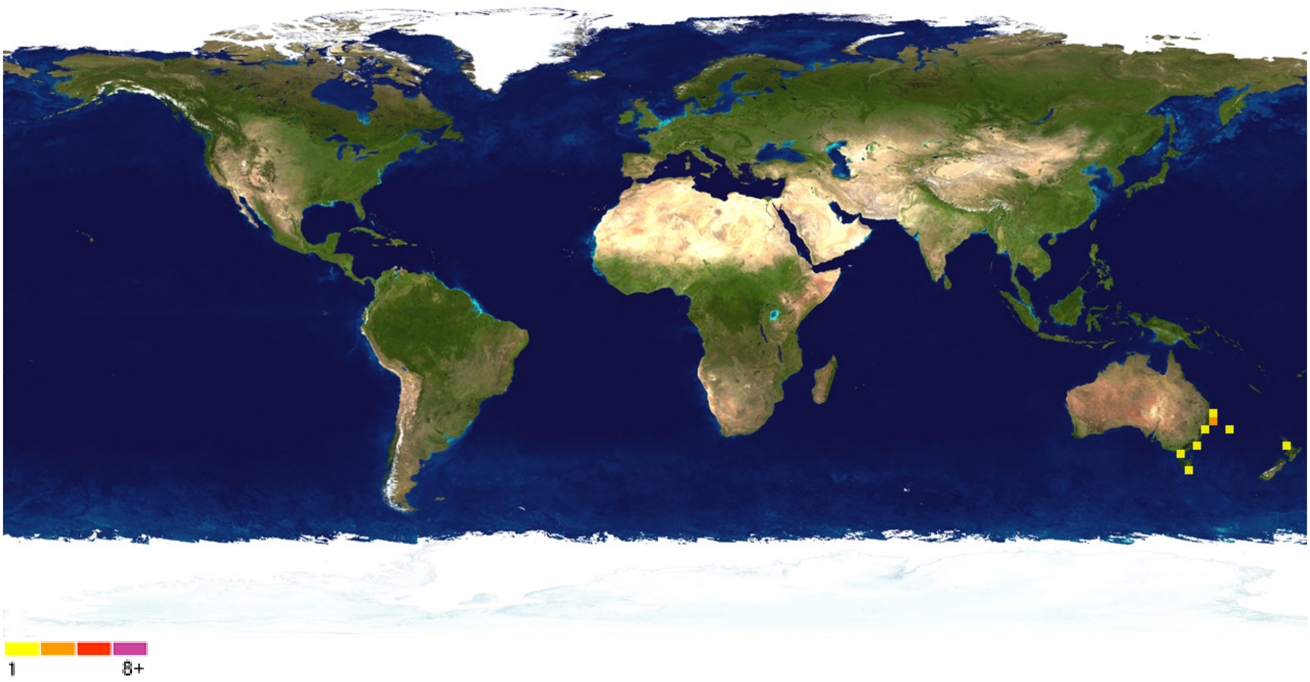

DATABASES

Public Data Portal
Taxonomy Browser
Publications
Primers

RESOURCES

Citing BOLD
News and Events
Data Releases

ORGANIZATION

About Us
Contact Us
News & Events

PARTNERS

iBOL
CBG
CCDB
GenBank
EOL
GBIF

UNIVERSITY of GUELPH

Copyright BOLD © 2014-2019
