# Supplementary material for: Could do better! A high school market survey of fish labelling in Sydney, Australia, using DNA barcodes
Source: PeerJ. 2019 Jun 14;7:e7138. doi: 10.7717/peerj.7138 (PMC6573807; doi:10.7717/peerj.7138)
Supplement: Supplemental Information 4 [file peerj-07-7138-s004.pdf]

IDENTIFICATION ENGINE: RESULTS

Results Summary

**Query ID**	**Best ID**	**Search DB**	**Tree**	**Top %**	**Graph**	**Low %**
SDP331062-16|KX781879|SGS224|Thunnus albacares|COI-5P	*Thunnus albacares*	COI SPECIES DATABASE		100.00	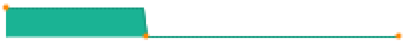	99.85

Query: SDP331062-16|KX781879|SGS224|Thunnus albacares|COI-5P
Top Hit: Chordata Actinopterygii - Scombriformes - *Thunnus albacares* (100%)

Search Result:

A species level match could not be made, the queried specimen is likely to be one of the following:

Thunnus albacares
Thunnus obesus
Thunnus atlanticus

For a hierarchical placement - a neighbor-joining tree is provided:

TREE BASED IDENTIFICATION

Identification Summary

Taxonomic Level	Taxon Assignment	Probability of Placement (%)
Phylum	Chordata	100
Class	Actinopterygii	100
Order	Scombriformes	100
Family	Scombridae	100
Genus	*Thunnus*	100

Similarity Scores of Top 99 Matches

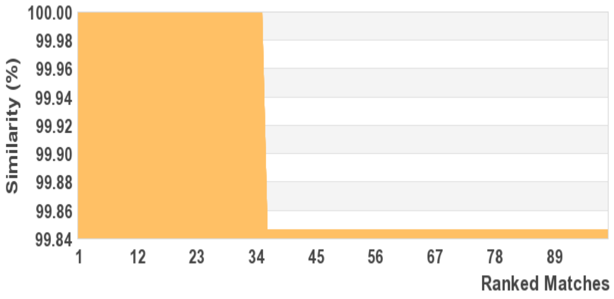

Display:

Top 20 ▼

Top 20 Matches

Phylum	Class	Order	Family	Genus	Species	Subspecies	Similarity (%)	Status
Chordata	Actinopterygii	Scombriformes	Scombridae	*Thunnus*	*albacares*		100	Published
Chordata	Actinopterygii	Scombriformes	Scombridae	*Thunnus*	*albacares*		100	Published
Chordata	Actinopterygii	Scombriformes	Scombridae	*Thunnus*	*albacares*		100	Published
Chordata	Actinopterygii	Scombriformes	Scombridae	*Thunnus*	*albacares*		100	Published
Chordata	Actinopterygii	Scombriformes	Scombridae	*Thunnus*	*albacares*		100	Published
Chordata	Actinopterygii	Scombriformes	Scombridae	*Thunnus*	*albacares*		100	Published
Chordata	Actinopterygii	Scombriformes	Scombridae	*Thunnus*	*albacares*		100	Published
Chordata	Actinopterygii	Scombriformes	Scombridae	*Thunnus*	*albacares*		100	Published

Chordata	Actinopterygii	Scombriformes	Scombridae	*Thunnus*	*albacares*	100	Published
Chordata	Actinopterygii	Scombriformes	Scombridae	*Thunnus*	*albacares*	100	Published
Chordata	Actinopterygii	Scombriformes	Scombridae	*Thunnus*	*albacares*	100	Published
Chordata	Actinopterygii	Scombriformes	Scombridae	*Thunnus*	*albacares*	100	Published
Chordata	Actinopterygii	Scombriformes	Scombridae	*Thunnus*	*albacares*	100	Published
Chordata	Actinopterygii	Scombriformes	Scombridae	*Thunnus*	*albacares*	100	Private
Chordata	Actinopterygii	Scombriformes	Scombridae	*Thunnus*	*albacares*	100	Private
Chordata	Actinopterygii	Scombriformes	Scombridae	*Thunnus*	*albacares*	100	Private
Chordata	Actinopterygii	Scombriformes	Scombridae	*Thunnus*	*albacares*	100	Private
Chordata	Actinopterygii	Scombriformes	Scombridae	*Thunnus*	*albacares*	100	Private
Chordata	Actinopterygii	Scombriformes	Scombridae	*Thunnus*	*albacares*	100	Private
Chordata	Actinopterygii	Scombriformes	Scombridae	*Thunnus*	*albacares*	100	Private

Sampling Sites For Top Hits (>98% Match)

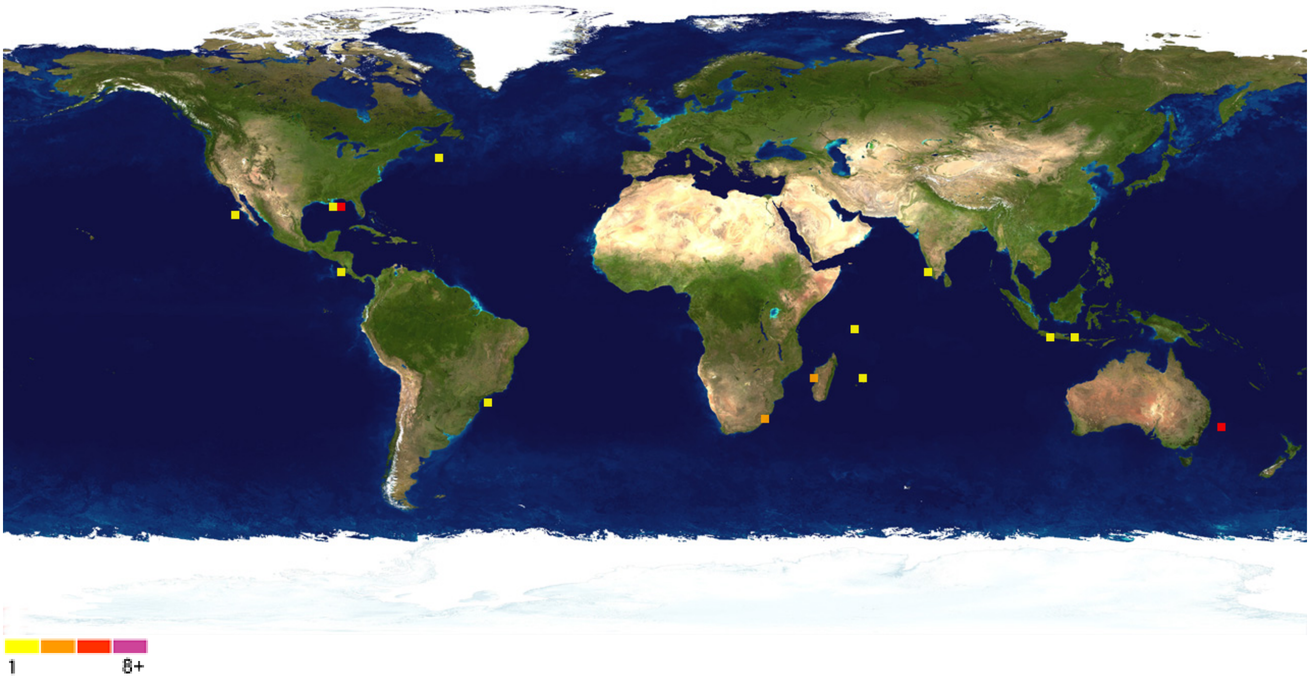

DATABASES

Public Data Portal
Taxonomy Browser
Publications
Primers

RESOURCES

Citing BOLD
News and Events
Data Releases

ORGANIZATION

About Us
Contact Us
News & Events

PARTNERS

iBOL
CBG
CCDB
GenBank
EOL
GBIF

Copyright BOLD © 2014-2019
